# Dual Inhibition of DKC1 and MEK1/2 Synergistically Restrains the Growth of Colorectal Cancer Cells

**DOI:** 10.1002/advs.202004344

**Published:** 2021-03-15

**Authors:** Guangyan Kan, Ziyang Wang, Chunjie Sheng, Gong Chen, Chen Yao, Yizhi Mao, Shuai Chen

**Affiliations:** ^1^ State Key Laboratory of Oncology in South China Collaborative Innovation Center for Cancer Medicine Sun Yat‐sen University Cancer Center Guangzhou Guangdong 510060 P. R. China; ^2^ Department of Colorectal Surgery State Key Laboratory of Oncology in South China Collaborative Innovation Center for Cancer Medicine Sun Yat‐sen University Cancer Center Guangzhou Guangdong 510060 P. R. China

**Keywords:** dyskerin pseudouridine synthase 1, MEK1/2, pyrazofurin, ribosomal protein, trametinib

## Abstract

Colorectal cancer, one of the most commonly diagnosed cancers worldwide, is often accompanied by uncontrolled proliferation of tumor cells. Dyskerin pseudouridine synthase 1 (DKC1), screened using the genome‐wide RNAi strategy, is a previously unidentified key regulator that promotes colorectal cancer cell proliferation. Enforced expression of DKC1, but not its catalytically inactive mutant D125A, accelerates cell growth in vitro and in vivo. DKC1 knockdown or its inhibitor pyrazofurin attenuates cell proliferation. Proteomics, RNA immunoprecipitation (RIP)‐seq, and RNA decay analyses reveal that DKC1 binds to and stabilizes the mRNA of several ribosomal proteins (RPs), including *RPL10A*, *RPL22L1*, *RPL34*, and *RPS3*. DKC1 depletion significantly accelerates mRNA decay of these RPs, which mediates the oncogenic function of DKC1. Interestingly, these DKC1‐regulated RPs also interact with HRAS and suppress the RAS/RAF/MEK/ERK pathway. Pyrazofurin and trametinib combination synergistically restrains colorectal cancer cell growth in vitro and in vivo. Furthermore, DKC1 is markedly upregulated in colorectal cancer tissues compared to adjacent normal tissues. Colorectal cancer patients with higher DKC1 expression has consistently poorer overall survival and progression‐free survival outcomes. Taken together, these data suggest that DKC1 is an essential gene and candidate therapeutic target for colorectal cancer.

## Graphical abstract

The study identified Dyskerin pseudouridine synthase 1 (DKC1) is upregulated in colorectal cancer and stabilizes the mRNA of several ribosomal proteins (RPs) to promote cancer progression. Besides, DKC1 inhibition induces unexpected activation of the oncogenic RAS/RAF/MEK/ERK pathway through the removal of RPs’ inhibition on HRAS. The pyrazofurin (DKC1 inhibitor) and trametinib (MEK inhibitor) combination synergistically restrains colorectal cancer cell growth.

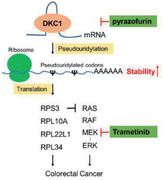

## 1 Introduction

Colorectal cancer (CRC) is the third most commonly diagnosed cancer and has the third highest rate of disease mortality among cancer types.^[^ ^1^ ^]^ It is a multifactorial cancer with various genetic and epigenetic aberrations.^[^ ^2^, ^3^ ^]^ Early screening with colonoscopies and the understanding of molecular mechanisms have contributed to decreases in colorectal cancer occurrence and mortality. For example, antibodies targeting vascular endothelial growth factor receptor (VEGFR) and epidermal growth factor receptor (EGFR) have been developed and widely used to treat CRC.^[^ ^4^, ^5^, ^6^ ^]^ Further exploration of the genes and signaling pathways involved in colorectal cancer would help to identify new prognostic and therapeutic targets.

The dyskerin pseudouridine synthase 1 (*DKC1*) gene was identified mutated in dyskeratosis congenita and encodes the evolutionarily conserved RNA‐binding protein dyskerin.^[^ ^7^, ^8^ ^]^ Dyskerin is a component of telomerase ribonucleoprotein that associates with three highly conserved proteins (NOP10, NHP2, and GAR1) and binds directly to telomerase RNA component (TERC) to maintain the telomerase activity.^[^ ^9^, ^10^ ^]^ The dyskerin protein complex also participates in forming small nucleolar ribonucleoprotein (snoRNP) complexes, which bind to H/ACA small nucleolar RNAs. Dyskerin acts as an RNA‐guided pseudouridine synthase, catalyzing the isomerization of uridine (U) nucleosides to pseudouridine (Ψ) nucleosides in its target RNAs,^[^ ^10^, ^11^ ^]^ which include ribosomal RNAs (rRNAs), small nuclear RNAs (snRNAs), mRNAs, snoRNAs, and long noncoding RNAs.^[^ ^12^, ^13^ ^]^ In addition, recent reports showed that dysregulated expression of DKC1 in various human cancer types altered cancer cell growth or metastasis and is associated with patient prognosis.^[^ ^14^, ^15^, ^16^ ^]^

In this study, we identified DKC1 as an essential top‐scoring gene in colorectal cancer via genome‐wide shRNA screening. DKC1 binds to and stabilizes the mRNA of some ribosomal proteins in a manner dependent on its pseudouridine synthase activity, thus promoting colorectal cancer progression in vitro and in vivo. The DKC1‐targeted ribosomal proteins interact with and inhibit HRAS, thus attenuating the downstream RAF/MEK/ERK pathway. The combination of the DKC1 inhibitor pyrazofurin (PF) and the MEK1/2 inhibitor trametinib synergistically suppressed colorectal cancer growth. Moreover, higher expression of DKC1 in colorectal cancer is associated with poorer prognosis, which suggests that DKC1 is a candidate therapeutic target for CRC.

## 2 Results

### 2.1 DKC1 Accelerates Colorectal Cancer Cell Proliferation

**Figure 1:**
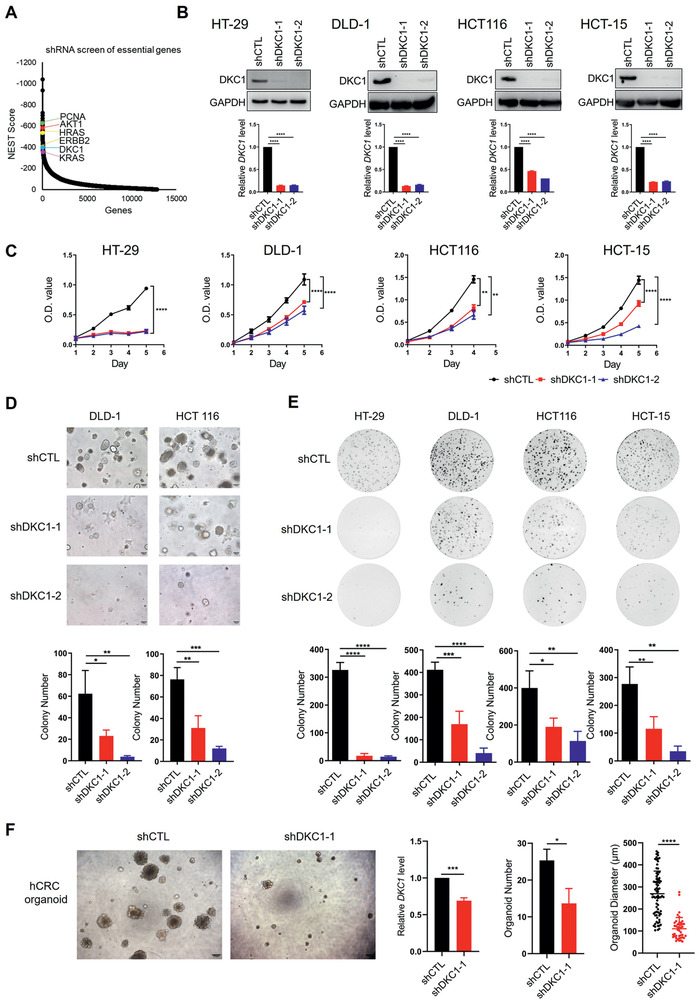
shRNA library screening identifies DKC1 promoting the proliferation of colorectal cancer cells. A) Negative selection of essential genes via NEST. B) Expression of DKC1 in colorectal cancer cell lines after lentiviral transduction of DKC1 shRNA or nonspecific shRNA (shCTL) by western blot (top) or qPCR (bottom). C) The cell growth curves of DKC1 knockdown or control colorectal cancer cells represent the O.D. values obtained from three independent CCK‐8 assays (mean ± standard deviation (SD)). D) 3D Matrigel colony formation assays of DKC1 knockdown and control cells. The top panel shows representative 3D Matrigel colony formation images (100×; the scale bar indicates 100 µm); the bottom panel shows bar charts indicating the number of colonies per well (mean ± SD). E) Colony formation assays of DKC1 knockdown and control cells. The top panel shows representative colony formation images; the bottom panel shows a bar graph of the number of colonies per well (mean ± SD). F) The left panel shows representative images of DKC1 knockdown or control human colorectal cancer (hCRC) organoids (scale bar: 200 µm). The right panel shows bar charts of *DKC1* expression and organoid number and size (mean ± SD). *P < 0.05, **P < 0.01, ***P < 0.001, ****P < 0.0001 ((B, D, E) one‐way ANOVA with Bonferroni correction, (C) two‐way ANOVA with Bonferroni correction, (F) two‐sided Student's *t*‐test).

To identify essential genes involved in the progression of colorectal cancer, we developed a high‐throughput shRNA library screening strategy.^[^ ^17^, ^18^ ^]^ The Network Essentiality Scoring Tool (NEST) was used for the negative selections.^[^ ^19^ ^]^ Several candidate proliferation‐related genes, including *DKC1* and five well‐known tumor growth‐associated genes (*PCNA*, *AKT1, HRAS*, *ERBB2*, and *KRAS*), were identified as top hits (**Figure** **1**A). To validate the screening results, we constructed CRC cell lines with stable DKC1 knockdown (Figure 1B). DKC1 silencing significantly decreased the growth of HT‐29, DLD‐1, HCT116, and HCT‐15 cells (Figure 1C) and the colony numbers of DLD‐1 and HCT116 cells in a 3D Matrigel growth assay (Figure 1D). The colony formation abilities of HT‐29, DLD‐1, HCT116, and HCT‐15 cells were also greatly reduced by DKC1 depletion (Figure 1E). In addition, DKC1 knockdown significantly reduced human colorectal cancer (hCRC) organoid number and size (Figure 1F).

### 2.2 Pseudouridine Synthase Activity is Indispensable for DKC1's Oncogenic Function

**Figure 2:**
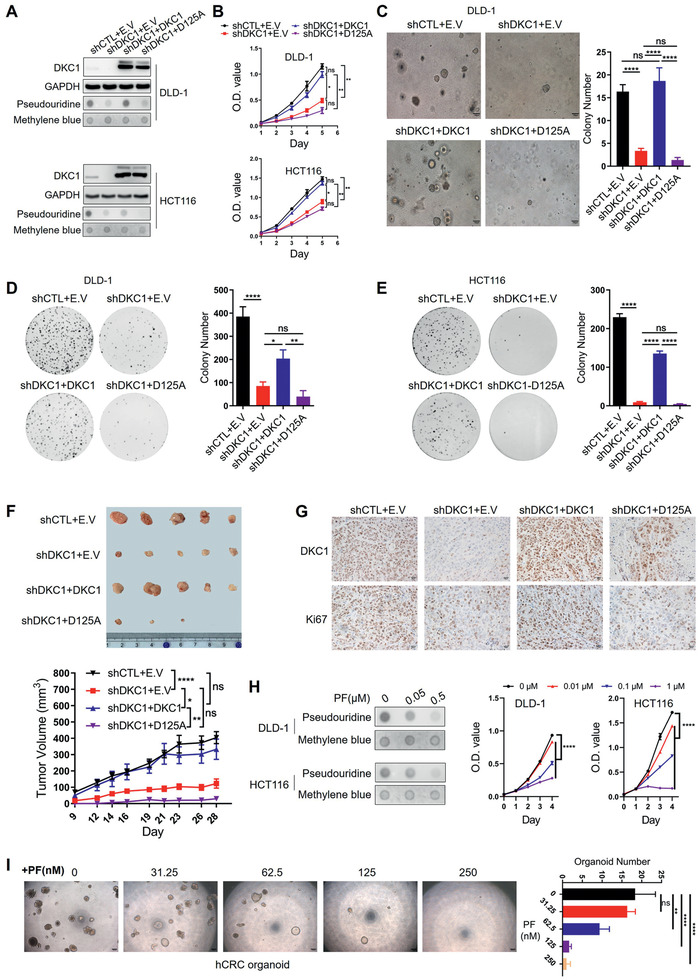
DKC1 promotes colorectal cancer cell growth via its pseudouridine synthase activity. A) Immunoblot detecting DKC1 expression and dot blots monitoring cellular pseudouridine levels in control and DKC1 knockdown DLD‐1 and HCT116 cells with rescued expression of wild‐type DKC1 or mutant DKC1 (D125A). B–E) Results of cell growth curve (B), 3D Matrigel assays (C), and colony formation (D–E) of DLD‐1 or HCT116 cells with DKC1 knockdown and rescued expression of wild‐type DKC1 or D125A from three independent experiments (mean ± SD). F–G) Subcutaneous tumor models were established with control or DKC1 knockdown DLD‐1 cells with or without rescued expression of wild‐type DKC1 or D125A. F) The top panel shows tumor images after 4 weeks of injection. The bottom panel shows tumor volumes recorded at the indicated times. G) Immunostaining of DKC1 and Ki67 in the indicated tumors. Scale bar: 20 µm. H) The effects of the DKC1 inhibitor pyrazofurin (PF) on overall pseudouridine levels in DLD‐1 and HCT116 cells by dot blots (left) and cell growth (right). I) The effects of the DKC1 inhibitor PF on human colorectal cancer (hCRC) organoids. The right panel shows representative images of hCRC organoid treated with the indicated concentration of PF. Scale bar: 200 µm. The right panel shows bar charts indicating organoid number (mean ± SD). E.V: empty vector. *P < 0.05, **P < 0.01, ****P < 0.0001, ns: no significance ((B, F, H) two‐way ANOVA with Bonferroni correction, (C, D, E, I) one‐way ANOVA with Bonferroni correction).

Since DKC1 is a member of the pseudouridine synthase family, we next examined whether the oncogenic function of DKC1 is dependent on its synthase activity. As the aspartic acid (D) at position 125 in the TruB domain is crucial for the pseudouridine synthase activity of DKC1 (Figure S1, Supporting Information),^[^ ^20^, ^21^ ^]^ we reintroduced lentiviral expression vector containing wild‐type DKC1 or a catalytically inactive DKC1 (D125A) into DLD‐1 and HCT116 cells with stable DKC1 knockdown (**Figure** **2**A). The total RNA pseudouridine level was decreased in shDKC1 cells and was restored by wild‐type DKC1 but not D125A (Figure 2A). The cell proliferation, 3D Matrigel growth and colony formation of both cell lines were markedly reduced with DKC1 knockdown and were restored by wild‐type DKC1 but not D125A (Figure 2B‐E). In accordance with the above in vitro studies, enforced expression of wild‐type DKC1, but not D125A, restored the tumor growth and Ki67 levels of DKC1‐silenced DLD‐1 cells in vivo (Figure 2F,G). Furthermore, treatment of the DKC1 inhibitor pyrazofurin (PF)^[^ ^22^ ^]^ significantly decreased the total RNA pseudouridine content and the growth of DLD‐1 and HCT116 cells (Figure 2H) and the formation of human CRC organoids (Figure 2I). These data demonstrate that the pseudouridine synthase activity of DKC1 is critical for its oncogenic function.

### 2.3 Proteomic Analysis Identifies Candidate Downstream Targets of DKC1

**Figure 3:**
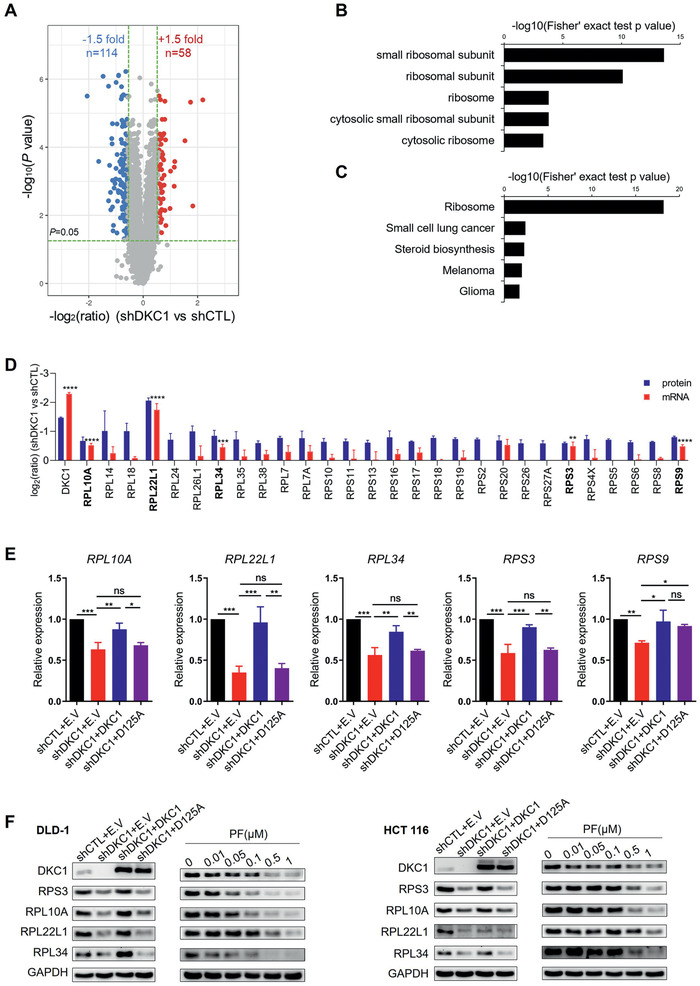
Proteomic analysis reveals that DKC1 elicits ribosomal protein expression. A) Volcano plot of differentially expressed proteins obtained from proteomic analysis of triplicate samples of DKC1 knockdown DLD‐1 cells and control cells. A total of 114 downregulated proteins and 58 upregulated proteins were included. B,C) GO enrichment analysis for the cellular component category (B) and KEGG enrichment analysis (C) of differentially expressed proteins. D) Comparison of the mRNA abundance of 28 ribosomal proteins measured by qRT‐PCR and their protein levels measured by proteomic analysis of DKC1 knockdown DLD‐1 cells and control cells (All proteins with *P* < 0.05). E) qRT‐PCR assays evaluating the mRNA levels of the indicated genes in control and DKC1 knockdown DLD‐1 cells with or without enforced expression of wild‐type DKC1 or the DKC1 mutant (D125A) from three independent experiments (mean ± SD). F) Immunoblots assessing the abundance of the indicated ribosomal proteins in control and DKC1 knockdown DLD‐1 and HCT116 cells with or without enforced expression of wild‐type DKC1 or D125A and PF‐treated DLD‐1 and HCT116 cells (48 h). E.V: empty vector. *P < 0.05, **P < 0.01, ***P < 0.001, ****P < 0.0001, ns: no significance ((D) two‐sided Student's *t*‐test, (E) one‐way ANOVA with Bonferroni correction).

To identify candidate downstream targets of DKC1, we next compared the proteome between control and DKC1‐silenced DLD‐1 cells. A total of 172 proteins were differentially expressed (fold change > 1.5, and *P* < 0.05), of which 58 were upregulated and 114 were downregulated (**Figure** **3**A). Gene Ontology (GO) and Kyoto Encyclopedia of Genes and Genomes (KEGG) analyses suggested that most of the differentially expressed proteins belonged to the ribosomal protein catalogue (Figure 3B,C). Interestingly, all the 28 differentially expressed ribosomal proteins were downregulated in the DKC1 knockdown group (Figure 3D; Table S1, Supporting Information). We then validated the expression of these 28 ribosomal proteins in control and DKC1‐knockdown DLD‐1 cells by quantitative RT‐PCR (qRT‐PCR) (Figure 3D). The protein (proteomic data) and mRNA abundances of five ribosomal proteins (RPL10A, RPL22L1, RPL34, RPS3, RPS9) were decreased in DKC1‐silenced cells (Figure 3D). Reintroduction of wild‐type DKC1, but not the D125A mutant, rescued the expression of four ribosomal proteins (*RPL10A*, *RPL22L1*, *RPL34*, and *RPS3*) in DKC1‐silenced DLD‐1 cells (Figure 3E). Similar expression patterns were also observed at the protein level (Figure 3F). In addition, the DKC1 inhibitor PF suppressed the expression of RPL10A, RPL22L1, RPL34, and RPS3 in DLD‐1 and HCT116 cells (Figure 3F). The findings demonstrate that DKC1 increases the abundances of some ribosomal proteins in a manner dependent on its pseudouridine synthase activity.

### 2.4 DKC1 Binds to and Stabilizes the mRNA of Target Ribosomal Proteins

**Figure 4:**
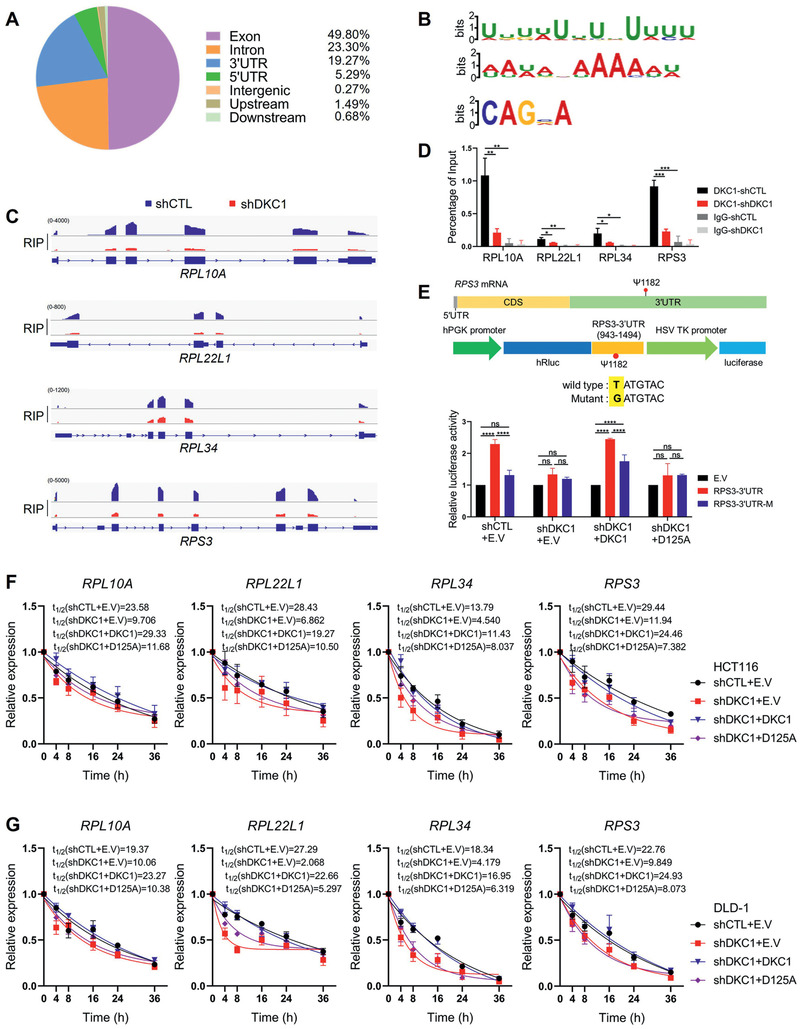
DKC1 binds to and stabilizes the mRNA of downstream ribosomal proteins. A) Distribution of peaks enriched by DKC1 on the genome in DLD‐1 cells. B) Three new DKC1 binding motifs were discovered in DLD‐1 shCTL cells. C) Integrative Genomics Viewer (IGV) was used to visualize the distribution of reads on the indicated genes. D) Validation of the genome‐wide DKC1 enrichment results in by RIP‐qPCR from three independent experiments (means ± SD). E) Evaluation of the function of the reported pseudouridylation site (Ψ1182) in the RPS3 3′UTR. Top panel: Schematic diagram of *RPS3* mRNA. Middle panel: Schematic diagram of constructs used for the dual luciferase assay. At position Ψ1182, a substitution of T with G was used to abolish the potentially modified sites in DKC1. Bottom panel: The effects of the wild‐type RPS3 3′UTR and mutant RPS3 3′UTR (RPS3‐3′UTR‐M) on luciferase activity in DKC1 knockdown HCT116 cells with or without enforced expression of wild‐type DKC1 or D125A were evaluated with a dual luciferase assay system. F,G) HCT116 or DLD‐1 cells were treated with actinomycin D (5 µg mL^−1^) for the indicated time. The abundance of the indicated mRNAs in control and DKC1 knockdown cells with or without enforced expression of wild‐type DKC1 or D125A was monitored by qRT‐PCR at different time points, and mRNA decay curves were constructed and fit. Data in the graph are shown as the means ± SD from three independent experiments. E.V: empty vector. *P < 0.05, **P < 0.01, ***P < 0.001, ****P < 0.0001, ns: no significance ((D) two‐sided Student's *t*‐test, (E) two‐way ANOVA with Bonferroni correction).

We employed RNA immunoprecipitation with sequencing (RIP‐seq) to identify mRNA regions bound by DKC1 in DLD‐1 cells. In mature mRNA, most reads were located in exons and 3′UTRs, with few found in 5′UTRs (**Figure** **4**A), and three new DKC1 binding motifs were identified (Figure 4B). The DKC1 binding efficiency for the abovementioned four target ribosomal proteins (*RPL10A*, *RPL22L1*, *RPL34*, and *RPS3*) was decreased in DKC1 knockdown cells compared with control cells (Figure 4C). RIP‐qPCR confirmed the enrichment of DKC1 on the mRNAs of these four ribosomal proteins, which was markedly decreased in DKC1 knockdown cells (Figure 4D).

Subsequently, we examined the role of DKC1 on one reported pseudouridine site (Ψ1182) in the 3′UTR of RPS3 mRNA that was discovered by N_3_‐CMC‐enriched pseudouridine sequencing (CeU‐seq).^[^ ^23^ ^]^ First, RIP‐qPCR confirmed the enrichment of DKC1 on the RPS3‐3′UTR, which was decreased in DKC1 knockdown cells and was restored in wild‐type DKC1 or D125A reintroduced HCT116 cells, with the enrichment significantly lower in D125A group (Figure S2, Supporting Information). A dual luciferase reporter system containing wild‐type RPS3‐3′UTR or an RPS3‐3′UTR mutant (RPS3‐3′UTR‐M) in which the T of the Ψ‐modified site was substituted with a G was introduced into DKC1 knockdown HCT116 cells with or without enforced expression of wild‐type DKC1 or D125A and control cells. The wild‐type RPS3‐3′UTR, but not RPS3‐3′UTR‐M, enhanced luciferase activity in control cells but not in DKC1 knockdown cells and the ectopic expression of DKC1, but not D125A, could rescue the luciferase activity enhanced by wild‐type RPS3‐3′UTR (Figure 4E). Since multiple studies have revealed the association between RNA modification and mRNA decay, this result suggested that the pseudouridine site Ψ1182 is probably crucial for DKC1 to stabilize *RPS3* mRNA. We next measured the mRNA stability of the four target ribosomal proteins (*RPL10A*, *RPL22L1*, *RPL34*, and *RPS3*). We found that the mRNA half‐life was significantly decreased in HCT116 cells and DLD‐1 cells with DKC1 downregulation compared with control cells, and reintroduction of wild‐type DKC1, but not the D125A mutant, rescued the mRNA half‐life in both cells (Figure 4F,G). These data indicate that the enrichment of DKC1 on the mRNAs of these ribosomal proteins maintains their stability and eventually increases their protein abundance.

### 2.5 Ribosomal Proteins Mediate the Oncogenic Activity of DKC1

**Figure 5:**
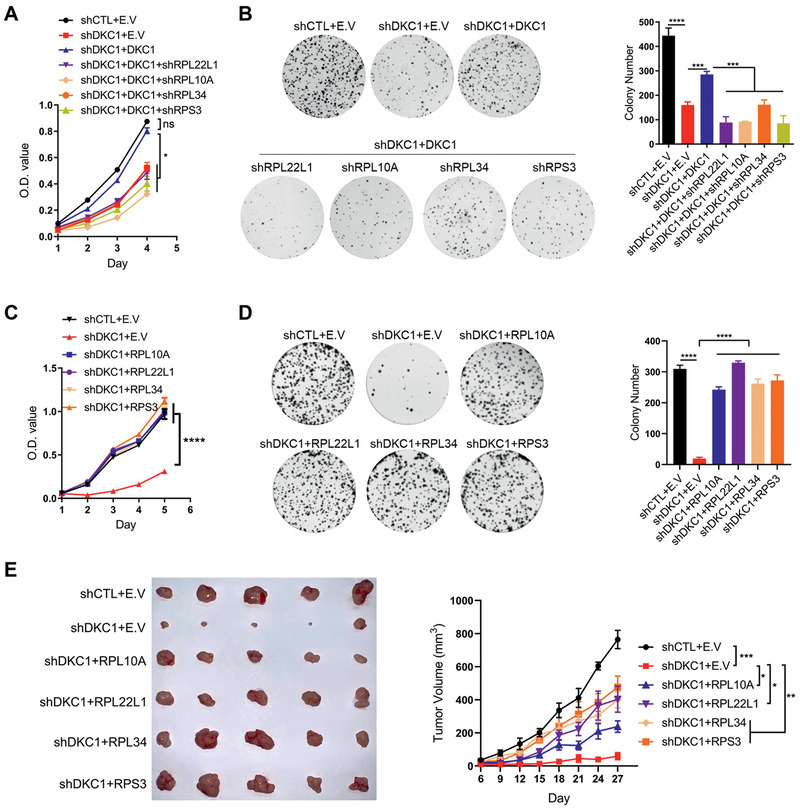
DKC1 promotes colorectal cancer cell growth by regulating ribosomal proteins. A,B) DLD‐1 cells with DKC1 knockdown were rescued with or without ectopic expression of DKC1 and were further transduced with lentiviral vectors carrying shRNA individually targeting the indicated ribosomal proteins. The cell growth curve (A) and colony formation (B) of these cells were assessed. (C‐D) DLD‐1 cells with DKC1 knockdown were transduced with lentiviral vectors encoding the indicated ribosomal proteins individually. C) The cell growth curve and D) colony formation of these cells and control cells were monitored. E) DLD‐1 cells with DKC1 knockdown were transduced with lentiviral vectors individually encoding the indicated ribosomal proteins. A total of 1.5 × 10^6^ of these cells and control DLD‐1 cells were subcutaneously injected into BALB/c nude mice. Tumor images after 4 weeks of injection (left). Tumor volumes recorded at the indicated times (right). E.V: empty vector. *P < 0.05, **P < 0.01, ***P < 0.001, ****P < 0.0001, ns: no significance ((A, C, E) two‐way ANOVA with Bonferroni correction, (B, D) one‐way ANOVA with Bonferroni correction).

As DKC1 accelerated colorectal cancer cell growth, we further explored whether this function is mediated by DKC1‐targeted ribosomal proteins. We first constructed HCT116 and DLD‐1 cells with individual knockdown of RPL10A, RPL22L1, RPL34, or RPS3 (Figure S3A,E, Supporting Information). The growth and colony formation abilities of both cell lines were robustly decreased by individual knockdown of each ribosomal proteins (Figure S3B–D,F–H, Supporting Information). Next, we performed a series of rescue experiments to evaluate whether these ribosomal proteins are required for the oncogenic function of DKC1. We knocked down RPL22L1, RPL10A, RPL34, or RPS3 individually in DKC1‐silenced DLD‐1 or HCT116 cells with or without enforced DKC1 expression (Figure S4A, Supporting Information). The capability of ectopically expressed DKC1 to promote the proliferation and colony formation of both cell lines was largely abolished with the depletion of each ribosomal protein (**Figure** **5**A,B; Figure S4B,C, Supporting Information). In another assay, individual transduction of RPL22L1, RPL10A, RPL34, and RPS3 markedly increased the growth and colony formation of DKC1‐silenced DLD‐1 and HCT116 cells (Figure 5C,D; Figure S4D–F, Supporting Information). A similar phenomenon was also observed in the xenograft tumors, wherein DKC1 silencing did not affect the tumor‐promoting function of these ribosomal proteins in DLD‐1 cells in vivo (Figure 5E). These data demonstrated that RPL22L1, RPL10A, RPL34, and RPS3 function downstream of DKC1 and are indispensable for DKC1 to promote CRC progression.

### 2.6 Combination of PF and Trametinib Synergistically Suppressed Colorectal Cancer Growth

**Figure 6:**
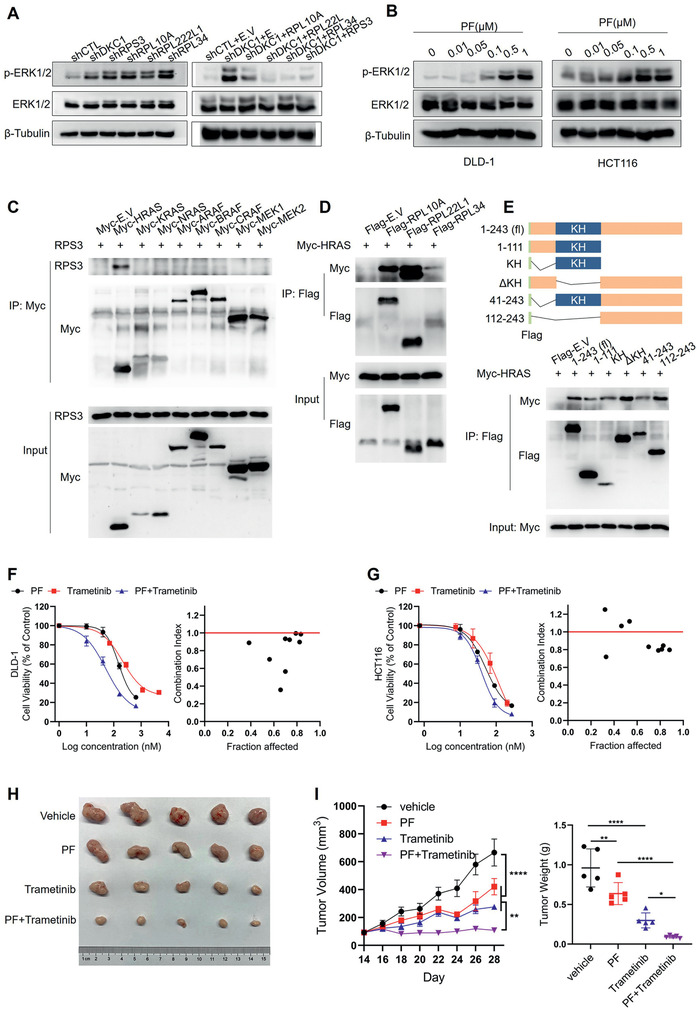
Combination of pyrazofurin and trametinib synergistically suppressed colorectal cancer. A) Immunoblot indicating the total expression levels and phosphorylation levels of ERK1/2 in DLD‐1 cells with knockdown of DKC1 or the indicated ribosomal proteins (left) or in DKC1‐silenced DLD‐1 cells with ectopic expression of DKC1 or the indicated ribosomal proteins (right). B) The effects of PF (48 h) on the phosphorylation levels of ERK1/2 in DLD‐1 and HCT116 cells by immunoblot. C) Lysates from HEK293T cells co‐transfected with plasmids expressing Myc‐tagged E.V, HRAS, KRAS, NRAS, ARAF, BRAF, CRAF, MEK1, and MEK2, along with RPS3 were immunoprecipitated with an anti‐Myc antibody and subjected to immunoblot analysis with an anti‐RPS3 antibody and anti‐Myc antibody. D) Lysates from HEK293T cells cotransfected with plasmids expressing Flag‐tagged E.V, RPL10A, RPL22L1, and RPL34, along with Myc‐HRAS were immunoprecipitated with an anti‐Flag antibody and subjected to immunoblot analysis with an anti‐Flag antibody and anti‐Myc antibody. E) (top) Schematic diagram showing the truncated RPS3. (bottom) Lysates from HEK293T cells cotransfected with plasmids expressing Flag‐tagged E.V, and the truncated RPS3, along with Myc‐HRAS were immunoprecipitated with an anti‐Flag antibody and subjected to immunoblot analysis with an anti‐Flag antibody and anti‐Myc antibody. F,G) The combination of PF and trametinib treatment on DLD‐1 (F) and HCT116 cells (G). Cell viability was assessed by ATP assay and the combination index was examined by Calcusyn. H,I) The combination of PF and trametinib treatment on subcutaneous tumor models established with HCT116 cells. (H) Tumor images at day 28 post‐injection. (I) Tumor volumes were recorded at the indicated times (left), and tumor weights were measured after dissection(right). E.V: empty vector. *P < 0.05, **P < 0.01, ****P < 0.0001, ((I) left, two‐way ANOVA with Bonferroni correction; right, one‐way ANOVA with Holm–Šídák's correction)

It has been reported that RPS3 could reduce the phosphorylation level of ERK.^[^ ^24^ ^]^ To validate whether DKC1‐targeted ribosomal proteins function similarly in colorectal cancer cells, we examined the p‐ERK1/2 level in DLD‐1 cells with DKC1 or ribosomal protein knockdown. We found that DKC1 or its targeted ribosomal protein (RPL22L1, RPL10A, RPL34, and RPS3) knockdown increased the phosphorylation of ERK1/2 (**Figure** **6**A) and MEK1/2 (Figure S5, Supporting Information). Ectopic expression of these ribosomal proteins in DKC1 silenced‐DLD‐1 cells restored the level of ERK1/2 phosphorylation (Figure 6A). In addition, DKC1 inhibitor PF treatment also increased the level of ERK1/2 phosphorylation in DLD‐1 and HCT116 cells (Figure 6B). These results suggest that DKC1‐targeted ribosomal proteins are negative regulators of the RAS/RAF/MEK/ERK signal pathway.

Next, we performed co‐immunoprecipitation (Co‐IP) assays to investigate the mechanism of DKC1‐targeted ribosomal proteins in mediating the inhibition of ERK phosphorylation. The ectopically expressed RPS3 specifically interacts with HRAS, but not other detected proteins in the RAS/RAF/MEK/ERK signal pathway (Figure 6C). The Co‐IP assays also confirmed the binding of RPL10A, RPL22L1, and RPL34 to HRAS (Figure 6D). Further, we generated a series of RPS3 truncated mutations, and the Co‐IP assays indicated that the C‐terminus of RPS3 (112‐243) has a relatively stronger interaction with HRAS (Figure 6E). More importantly, a HRAS activation assay showed that the recombinant human RPS3 fused with His tag (His‐RPS3) inhibits HRAS directly in vitro and HRAS activation was increased in RPS3 knockdown cells (Figure S6, Supporting Information). These results suggest that the DKC1‐targeted ribosomal proteins bind to and inhibit HRAS activity, thus repressing the RAS/RAF/MEK/ERK signal pathway.

As described above, although PF treatment significantly inhibits colorectal cancer cell growth (Figure 2), an unexpected alternative activation of the RAS/RAF/MEK/ERK pathway was observed (Figure 6B; Figure S7, Supporting Information), which would antagonize the anti‐tumor effect of PF. The application of the MEK inhibitor trametinib thus could attenuate PF‐induced ERK1/2 phosphorylation in DLD‐1 and HCT116 cells (Figure S7, Supporting Information), and we wondered whether the combination of PF (DKC1 inhibitor) and trametinib (MEK inhibitor) could synergistically suppress tumor growth. The combination therapy showed less than 1 combination index (CI) value on DLD‐1 cells (Figure 6F) and less than 1 CI value except at some low doses of PF and trametinib on HCT116 cells (Figure 6G), indicating a synergistic inhibitory effect of these two compounds. Moreover, PF (5 mg per kg, every 3rd day) and trametinib (300 µg per kg, daily) combination therapy also showed higher anticancer efficacy against HCT116 xenograft in vivo (Figure 6H,I).

### 2.7 High Expression of DKC1 in CRC Tissues Predicts Poor Prognosis

**Figure 7:**
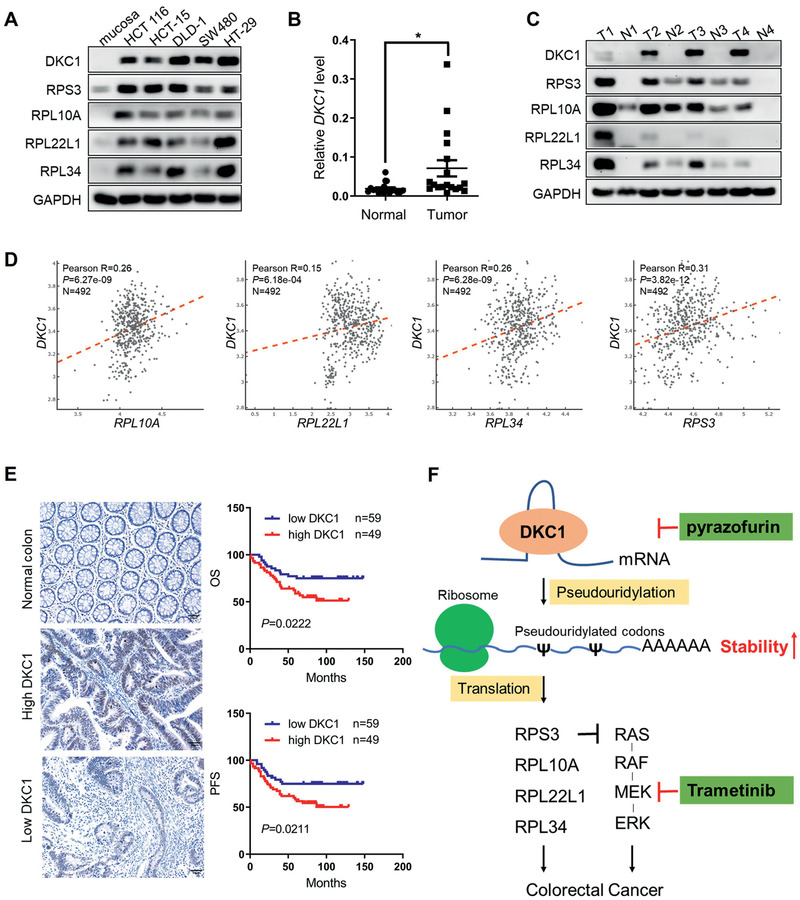
High expression of DKC1 in colorectal cancer predicts poor prognosis. A) Immunoblot indicating the expression of DKC1 and its downstream ribosomal proteins in colorectal cancer cell lines and normal mucosa. B) qRT‐PCR analysis evaluating the relative expression levels of *DKC1* mRNA in 18 pairs of human primary colorectal cancer tissues and normal mucosa. C) The expression of DKC1 and the four indicated ribosomal proteins in 4 pairs of human primary colorectal cancer tissues (T) and normal mucosa (N) was assessed by immunoblotting. D) Correlation analysis between the *DKC1* level and the mRNA abundance of the indicated ribosomal proteins in the TCGA colorectal cancer database. E) IHC analysis of 108 pairs of colorectal cancer and adjacent normal tissues. (Left) Representative IHC staining of DKC1 in the normal colon, high DKC1 expression and low DKC1 expression groups. Scale bar: 50 µm. (Right) Kaplan–Meier analysis of OS and PFS according to DKC1 expression in 108 colorectal cancer patients. F) A proposed model of the mechanism of pseudouridine synthase DKC1 to promote colorectal cancer cell proliferation and the rational of combination therapy using PF (DKC1 inhibitor) and trametinib (MEK1/2 inhibitor) to treat colorectal cancer. *P < 0.05, ((B) two‐sided Student's *t*‐test).

We examined DKC1 expression in human CRC cell lines and primary CRC tissues. The five examined CRC cell lines exhibited high expression of DKC1 and DKC1‐targeted ribosomal proteins (RPL10A, RPL22L1, RPL34, and RPS3), while normal mucosa had very low expression levels of these proteins (**Figure** **7**A). qRT‐PCR analysis of 18 pairs of human primary colorectal cancer tissues and adjacent normal tissues showed higher expression of DKC1 mRNA in cancerous tissues (Figure 7B). Western blots using another 4 pairs of tissues also revealed higher expression of DKC1 and ribosomal proteins in tumors than in normal tissues (Figure 7C). In addition, RNA‐seq data from The Cancer Genome Atlas (TCGA) confirmed that the abundance of *RPL10A*, *RPL22L1*, *RPL34*, and *RPS3* was positively correlated with *DKC1* level (Figure 7D). Finally, we investigated the prognostic relevance of the high expression of DKC1 in CRC patients. Immunohistochemical (IHC) staining of DKC1 was performed on 108 CRC tissues, and almost no DKC1 expression was detected in adjacent normal tissues (Figure 7E). Kaplan–Meier analysis revealed that patients with high DKC1 expression had worse overall survival (OS) and progression‐free survival (PFS) than those with low DKC1 expression (Figure 7E). These data suggest that DKC1 is highly expressed in CRC tissues and is associated with poor prognosis in CRC patients.

## 3 Discussion

RNA modifications play important roles in the posttranscriptional regulation of gene expression programs. In addition to regulating physiological cellular processes, RNA modifications contribute to cancer development by dysregulating RNA splicing, polyadenylation, stability, translation initiation, and gene expression.^[^ ^25^ ^]^ More than 170 RNA modifications have been identified to date, and pseudouridine is the most abundant modified nucleoside.^[^ ^26^ ^]^ Similar to other modified nucleosides, pseudouridine is generally a metabolic end product that cannot be salvaged and is excreted in urine.^[^ ^27^ ^]^ Elevated levels of pseudouridine have been observed in the urine of cancer patients, and urinary level of pseudouridine has been proposed as a diagnostic marker for various malignant neoplasms, including prostate cancer,^[^ ^28^ ^]^ liver cancer,^[^ ^29^ ^]^ gastric cancer, and colorectal cancer.^[^ ^30^ ^]^ The aberrant expression of RNA modification enzymes and the high rate of RNA turnover may be responsible for the elevated levels of urinary modified nucleosides in cancer patients.^[^ ^31^ ^]^ In accordance with these observations, we found that abnormally high expression of DKC1 altered pseudouridine patterns in CRC cells. DKC1 depletion and the DKC1 inhibitor pyrazofurin significantly decreased pseudouridine levels in CRC cells. Moreover, CRC patients with higher DKC1 expression have shorter OS and PFS, suggesting that DKC1 and pseudouridine levels could be used as valuable prognostic markers.

As the basic building blocks of ribosomes, ribosomal proteins play important roles in ribosome biogenesis and protein synthesis. Accumulating evidence has revealed that increased synthesis of ribosome biogenesis proteins sustains tumor cell growth and proliferation. Dysregulation of ribosomal protein expression is commonly observed in human cancers.^[^ ^32^ ^]^ Many studies have revealed that ribosomal proteins in cancer also have extraribosomal functions, such as mediating transcriptional regulation, DNA repair, and apoptosis.^[^ ^33^, ^34^ ^]^ For example, RPL10A is involved in cell proliferation and apoptosis,^[^ ^35^ ^]^ and RPL22L1 has been found to be overexpressed in ovarian cancer and to promote ovarian cancer metastasis via epithelial‐to‐mesenchymal transition.^[^ ^36^ ^]^ RPL34 knockdown was found to significantly inhibit esophageal cancer cell proliferation, migration, and invasion by regulating the PI3K/Akt signaling pathway.^[^ ^37^ ^]^ In addition RPS3, a non‐Rel subunit of the NF‐*κ*B complex,^[^ ^38^ ^]^ promotes hepatocarcinogenesis by stabilizing the mRNA of the oncogene silent information regulator 1 (SIRT1).^[^ ^39^ ^]^ Our study showed that these four ribosomal proteins (RPL10A, RPL22L1, RPL34, and RPS3) exhibited higher expression in CRC tissues than in adjacent normal tissues and that their mRNAs are bound by DKC1. It has been reported that pseudouridylation prolongs the half‐life of mRNA, and this function was achieved through two distinct mechanisms: (1) pseudouridine forms Watson–Crick base pairs with adenine (A) that have greater thermodynamic stability than U‐A pairs; 2) pseudouridine alters the recruitment of RNA‐binding proteins that regulate RNA processing, localization and/or stability.^[^ ^13^, ^40^, ^41^ ^]^ In this study, we found that DKC1 was mainly located in exons and 3′UTRs of the candidate target mRNAs. Knockdown DKC1 expression greatly reduced the stability of mRNAs encoding RPL10A, RPL22L1, RPL34, and RPS3, and enforced expression of the wild‐type DKC1, but not its catalytic dead mutant (D125A), rescued the half‐life of these mRNAs (Figure 4F,G). In addition, functional assays revealed that these ribosomal proteins function downstream of DKC1 to accelerate CRC cell proliferation. Targeting DKC1 by shRNA or its inhibitor PF decreased the expression of the abovementioned ribosomal proteins at both the mRNA and protein levels. This strategy makes these “undruggable” ribosomal proteins “druggable.”

Pyrazofurin (PF) has been tested in clinical trials in colorectal cancer as an orotidine 5′‐monophosphate (OMP) decarboxylase inhibitor, but it was ineffective due to the low response rate and toxicity.^[^ ^22^ ^]^ Based on our results, precision targeting of tumors with high DKC1 abundance may increase the effectiveness of PF in cancer therapy. In addition, we found that the abovementioned DKC1‐targeted ribosomal proteins (RPL10A, RPL22L1, RPL34, and RPS3) could attenuate another oncogenic pathway, namely, RAS/RAF/MEK/ERK signaling. As an example, RPS3 directly binds to HRAS and inhibits its GTPase activity. As a consequence, DKC1 inhibition (knockdown or PF treatment)‐induced unexpected activation of the tumor‐promoting RAS‐ERK pathway would largely counteract the antitumor effects. These phenomena, to a certain extent, explained the failure of PF in clinical trials. Our study also showed that trametinib (MEK inhibitor) could efficiently inhibit PF‐induced activation of RAS‐ERK signaling (Figure S7, Supporting Information). Based on these findings, dual inhibition of DKC1 and MEK1/2 synergistically suppressed the growth of colorectal cancer cells both in vivo and in vitro, which may be of benefit for future clinical trials to treat CRC patients with DKC1 overexpression (Figure 7F).

In summary, we identified DKC1 as an essential gene for colorectal cancer cell proliferation through genome‐wide RNAi screening. High expression levels of DKC1 promoted colorectal cancer progression by increasing the expression of ribosomal proteins in a pseudouridine synthase activity‐dependent manner. At the same time, DKC1‐targeted ribosomal proteins suppressed the RAS/RAF/MEK/ERK pathway via interaction with HRAS, and combination therapy with PF and trametinib exhibited synergistic anticancer efficacy in colorectal cancer cells. This work indicates that DKC1 is a candidate therapeutic target in colorectal cancer.

## 4 Experimental Section

##### NEST (Network Essentiality Scoring Tool) Analysis

The fold change of each shRNA abundance between Day 4 and Day 0 groups was calculated.^[^ ^17^, ^18^ ^]^ NEST negative selection was performed to identify the candidate tumor cell growth‐related genes.^[^ ^19^ ^]^ The examined log_2_(fold change) of several shRNAs was first merged for the same gene using the method of “top3” (mean of top three values by absolute). The candidate genes met the following criteria: (i) log_2_(fold change) ←0.5, (ii) FDR < 0.05, (iii) NEST score rank < 100.

##### Cell Culturing and Human Tissues

HT‐29, DLD‐1, HCT116, and HCT‐15 cells were obtained from American Type Culture Collection (ATCC) and maintained by following the culture instructions of ATCC. Twenty‐two pairs of colorectal cancer tissues and the matched normal tissues and 108 cases of paraffin‐embedded specimens were obtained from Sun Yat‐Sen University Cancer Center.

##### Generation of Stable Knockdown or Overexpression Cells

shRNA for DKC1 (shDKC1‐1, ‐2), RPL10A (shRPL10A‐1, ‐2), RPL22L1 (shRPL22L1‐1, ‐2), RPL34 (shRPL34‐1, ‐2), or RPS3 (shRPS3‐1, ‐2) was cloned into lentiviral pLKO.1 construct (Sigma‐Aldrich, St. Louis, USA). The sequences of the shRNA are provided in Table S2 (Supporting Information) and verifications of shRNAs for DKC1 being cloned into vector by sanger sequence are provided in Figure S8 (Supporting Information). The coding sequences of DKC1 and the DKC1 mutant (D125A) CDS with a substitution of aspartic acid (D) with alanine (A) at position 125, RPL10A, RPL22L1, RPL34, or RPS3 were cloned into lentiviral pCDH‐EF1‐T2A‐zeocin vector (System Bioscience, CA, USA). The virus particles were produced by following the lentivirus packaging protocol of Addgene. After shRNA lentivirus infection of colorectal cancer cells, puromycin (Thermo Fisher Scientific, Waltham, USA) selection was applied for at least 7 d to obtain stable knockdown cells. The overexpression lentivirus‐infected DKC1 stable knockdown cells underwent selection with zeocin (Thermo Fisher Scientific, Waltham, USA) for at least 7 d to obtain ectopically expressed cells.

##### Human Colorectal Cancer Organoid Culture and Transfection

Human colorectal cancer tissues were obtained from Sun Yat‐Sen University Cancer Center. The use of these samples was approved by the Sun Yat‐Sen University Cancer Center institutional review board. Isolation, generation, and culture of human colorectal cancer organoids were performed as described previously.^[^ ^42^ ^]^ shRNA for DKC1 (shDKC1‐1) was cloned into lentiviral pLKO‐Tet‐On vector. The lentivirus‐infected organoids were subjected to puromycin selection. Doxycycline (MedChemExpress, NJ, USA, # HY‐N0565) was used to induce shRNA expression. Quantitative RT‐PCR was used to detect knockdown efficiency, with 18s rRNA used as an internal control. The sequences of the primers are listed in Table S3 in the Supporting Information.

##### Cell Proliferation Assay

For the cell growth curve, 2500 cells were plated in one well of a 96‐well plate with at least three repeats for each cell, and Cell Counting Kit‐8 (CCK‐8, Dojindo, Tokyo, Japan) was used to perform continuous detection for 4–5 d. For the colony formation assay, 1000 cells were plated in one well of a 6‐well plate with at least three repeats for each cell and, 7–9 d later, were stained with crystal violet. Image J was used for colony number counting. To assay the effect of pyrazofurin (Sigma‐Aldrich, St. Louis, USA, #SML1502) on cell growth, 1500 cells were plated in one well of a 96‐well plate with at least three repeats for each condition, and the next day, pyrazofurin or vehicle was added. CCK‐8 was used to perform the continuous detection for 4–5 d.

##### 3D Matrigel Growth Assay

500 colorectal cancer cells in 25 µL Matrigel (Corning, AR, USA, #356 231) were plated in one well of a 24‐well plate, with three repeats for each cell. The cells were cultured for 7 d, and then images were taken using a microscope. The 40× images of each well were used for counting colony numbers.

##### Co‐IP and Immunoblot Assay

Cells were lysed with cell lysis buffer (Cell Signaling Technology, Boston, USA, #9803S) containing protease inhibitors cocktail and phosphatase inhibitors (Bimake, shanghai, China). For Co‐IP, after centrifugation, supernatants were collected and incubated with appropriate antibodies for 1 h at 4 °C, followed by protein G beads (Santa Cruz Biotechnology, CA, USA, #sc‐2002) overnight. After incubation, beads were washed with IP buffer. Immunoblot assays were performed with specific antibodies. The following antibodies were used for Co‐IP or immunoblot assay: DKC1 (Santa Cruz Biotechnology, CA, USA, #sc‐373956), RPL10A (Proteintech, Chicago, USA, #16681‐1‐AP), RPL22L1 (Immunoway, TX, USA, #YN1235), RPL34 (Affinity Biosciences, Cincinnati, OH, USA, #DF3708), RPS3 (Cell Signaling Technology, Boston, USA, #9538), Myc (RM1003, Ray Antibody, Beijing, China), Flag (Sigma, St. Louis, USA, #F1804), and His (Proteintech, Chicago, USA, #66005‐1‐Ig).

##### Dot Blot

2 µg total RNA in 2 µL water was denatured and loaded onto nylon membrane. After UV crosslinking, the membrane was probed with anti‐pseudouridine antibody (MBL, Nagoya, Japan, #D347‐3). After chemiluminescence detection, the membrane was stained with methylene blue as a loading control.

##### Subcutaneous Xenograft and Treatment

BABL/c female nude mice were obtained from Beijing Vital River Laboratory Animal Technology Company (Beijing, China). A total of 1.5 million cells were injected to 5–6‐week‐old mice subcutaneously. When tumors reached approximately 50 mm^3^, the mice were randomly assigned into four groups: the first group was treated with vehicle, the second group was treated with pyrazofurin (5 mg per kg, intraperitoneal injection, every 3rd day, Bioberry, DE, USA, #110 370), the third group was treated with trametinib (300 µg per kg, orally, daily, MedChemExpress, NJ, USA, #HY‐10999), and the fourth group was treated with both pyrazofurin and trametinib. Tumor length and width were measured every 2 d until the completion of treatment. Tumor volume was calculated by using the following formula: tumor volume = (length × width^2^)/2. At the end of experiment, the tumor weight was measured and the dissected tumors went for immunohistochemistry analysis.

##### Immunohistochemistry (IHC) and Survival Analysis

The following antibodies were used for immunohistochemistry: DKC1 (Santa Cruz Biotechnology, CA, USA, #sc‐373956) and Ki67 (BD Biosciences, Franklin, USA, #550 609). The DKC1 expression of human colorectal cancer tissues was interpreted independently by two pathologists. Two characteristics were used for scoring the expression of DKC1 in slices: overall stain intensity (with possible values ranging from 0 to 3) and a score representing the percentage of tumor cells that were stained (1, 0–25%; 2, 25–50%; 3, 50–75%, and 4, >75%). An IHC score was then calculated by multiplying the values of the two characteristics. Based on the IHC score, patients were divided into two groups: high DKC1 expression (IHC score ≥  4) and low DKC1 expression (IHC score < 4). Overall survival (OS) and progression‐free survival (PFS) were estimated using the Kaplan–Meier method, and the log‐rank test was used for statistical analysis.

##### Proteomic Analysis

DKC1 stable knockdown DLD‐1 cells and control cells in triplicate were lysed. The peptides obtained from trypsin digestion were labeled by using a TMT kit (Thermo Fisher Scientific, Waltham, USA). After LC‐MS/MS analysis, the MS/MS data were processed using Maxquant (v1.5.2.8) and searched against the Human SwissProt database (20387 entries), according to the following settings: (i) cleavage enzyme: Trypsin/P; (ii) missing cleavages: 2; (iii) mass tolerance for precursor ions: 20 ppm in First search and 5 ppm in Main search; (iv) mass tolerance for fragment ions: 0.02 Da; (v) minimum length of peptides: 7aa; (vi) quantitative method: TMT‐6plex; and (vii) FDR < 1%. The number of identified proteins was 5056, of which 3910 were quantifiable. Differentially expressed proteins were identified according to the criteria *P* value < 0.05 and |fold change| >1.5. Gene Ontology (GO) and Kyoto Encyclopedia of Genes and Genomes (KEGG) pathway enrichment analyses were performed to interpret the function of the differentially expressed proteins.

##### RNA Immunoprecipitation (RIP) Sequencing and RIP‐qPCR

Cells were harvested and ruptured with Lysis Buffer (Cell Signaling Technology, Boston, USA, #9803S) containing protease inhibitor cocktail (Thermo Fisher Scientific, Waltham, USA, #78 425) and RNase inhibitor (Thermo Fisher Scientific, Waltham, USA, #N8080119). RNA was fragmented into fragments of approximately 500bp by sonication. A 1/10 volume of the sonicated cell lysate was collected for input. The precipitated RNA with anti‐DKC1 antibody or mouse IgG was used for sequencing or qPCR assay. The clean sequencing data were aligned to the reference genome (hg19) using Tophat (v2.0.13). Integrative Genomics Viewer (IGV) was used to visualize the distribution of the uniquely mapped reads. The MEME and DREME tools were used for discovering motifs. The sequences of the primers used for RIP‐qPCR are listed in Table S3 in the Supporting Information.

##### mRNA Stability Assay

Colorectal cancer cells were treated with actinomycin D (5 µg mL^−1^, MedChemExpress, NJ, USA, #HY‐17559) for 0, 4, 8, 16, 24, and 36 h. The mRNA abundance of each time point was normalized to the mRNA abundance of 0 h. The time of half mRNA decay (*t*_1/2_) was calculated by nonlinear regression fitting (one phase decay) using GraphPad Prism software. The sequences of the primers are listed in Table S3 in the Supporting Information.

##### Quantitative RT‐PCR

The qRT‐PCR was performed by analyzing samples in triplicate. The results were normalized by the expression level of GAPDH as an internal control. The sequences of the primers for qRT‐PCR are listed in Table S3 in the Supporting Information.

##### Dual‐Luciferase Reporter Assay

psiCHECK‐2 vector was used for the dual‐luciferase reporter assay. The wild‐type RPS3‐3′UTR or mutant RPS3‐3′UTR (RPS3‐3′UTR‐M) was cloned into psiCHECK‐2 vector, and then the vector was transfected into DKC1 knockdown HCT116 cells or control cells respectively. The luciferase activities were measured using a Dual‐luciferase Reporter Assay System (Promega, Madison, USA, #E1910).

##### HRAS Activation Assay

The coding sequences of RPS3 were cloned into pET‐28a and the *E.coli* expressed recombinant human RPS3 fused with His tag (His‐RPS3) was purified and used for the HRAS activation assay. Follow the protocol to perform the HRAS activation assay (Cell Biolabs, CA, USA, # STA‐400‐H) for DLD‐1 cell lysates (3 mg) with or without His‐RPS3 and DLD‐1 cells with RPS3 knockdown or control cells.

##### Cell Viability Assay and Assessment of Combination Index

Cells were plated in 96‐well plates at a density of 1500 cells per well with at least three repeats for each condition. Cells were treated with vehicle, pyrazofurin (PF), trametinib or PF plus trametinib for 96 h. Then, ATP assays (Promega, Madison, USA, # G9241) were performed. The combination indexes (CIs) between PF and trametinib were calculated by Calcusyn. CI < 1 indicates a synergistic effect, CI = 1 indicates additive effect, and CI > 1 indicates antagonistic effect.

##### Statistical Analysis

GraphPad Prism was used to conduct the statistical analysis for most of the data unless indicated otherwise. Statistical significance was assessed by a two‐sided Student's *t*‐test from at least three independent repeats between two groups and one‐way analysis of variance (ANOVA) or two‐way ANOVA among multiple groups except when stated otherwise.

##### Ethics Declarations

The study with human clinical samples was approved by the Sun Yat‐Sen University Cancer Center institutional review board (approval numbers: GZR2019‐197 and GZR2020‐140). All animal studies were carried out according to the National Institute of Health Guide for the Care and Use of Laboratory Animals with the approval of Sun Yat‐Sen University Cancer Center Institutional Animal Care and Use Committee (approval number: L102012019220A).

## Conflict of Interest

The authors declare no conflict of interest.

## Author Contributions

G.K. and S.C. designed the research. G.K. performed most of the experiments. Z.W. and G.C. helped with the clinical samples. C.S., C.Y., and Y.M. provided technical assistance. S.C. conceived the study. G.K. and S.C. analyzed the data and wrote the manuscript.

## Supporting information

Supporting Information

## Data Availability

The datasets and computer code produced in this study are available in the following databases: Mass spectrometry proteomics data: PRIDE PXD018103 (http://www.ebi.ac.uk/pride/archive/projects/PXD018103). RIP‐seq data: Gene Expression Omnibus GSE147348 (https://www.ncbi.nlm.nih.gov/geo/query/acc.cgi?acc=GSE147348). Raw data of this study have been deposited in the Research Data Deposit database (http://www.researchdata.org.cn), and the accession number is RDDB2021000843.
