# Supplementary material for: Dual Inhibition of DKC1 and MEK1/2 Synergistically Restrains the Growth of Colorectal Cancer Cells
Source: Adv Sci (Weinh). 2021 Mar 15;8(10):2004344. doi: 10.1002/advs.202004344 (PMC8132060; doi:10.1002/advs.202004344)
Supplement: Supplementary file 1 — Supporting Information [file ADVS-8-2004344-s001.pdf]

© 2021 Wiley-VCH GmbH

ADVANCED SCIENCE
Open Access

Supporting Information

for *Adv. Sci.,* DOI: 10.1002/advs.202004344

Dual inhibition of DKC1 and MEK1/2 synergistically restrains the growth of colorectal cancer cells

*Guangyan Kan, Ziyang Wang, Chunjie Sheng, Gong Chen, Chen Yao, Yizhi Mao, Shuai Chen**

Supporting Information

Dual inhibition of DKC1 and MEK1/2 synergistically restrains the growth of colorectal cancer cells

Guangyan Kan, Ziyang Wang, Chunjie Sheng, Gong Chen, Chen Yao, Yizhi Mao, Shuai Chen*

These supporting information include:

1) Eight supplementary figures
2) Three supplementary tables

Supplementary Figures

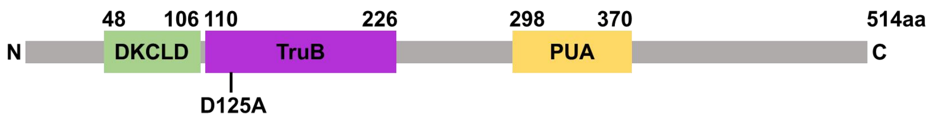

Figure S1. Schematic representation of the dyskerin domain structure. A substitution of aspartic acid (D) with alanine (A) at position 125 abolished dyskerin pseudouridine synthase activity, and the resulting mutant was used as a catalytically inactive DKC1 mutant (D125A).

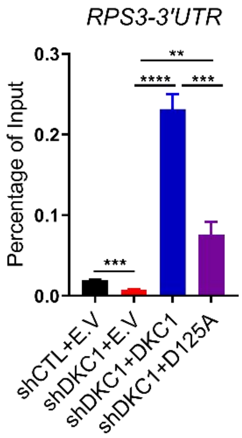

Figure S2. RIP-qPCR was performed to confirm the binding of DKC1 on RPS3-3′UTR in DKC1 silenced DLD-1 cells with enforced expression of DKC1 or D125A. **P<0.01, ***P<0.001, ****P<0.0001, two-sided Student's t-test.

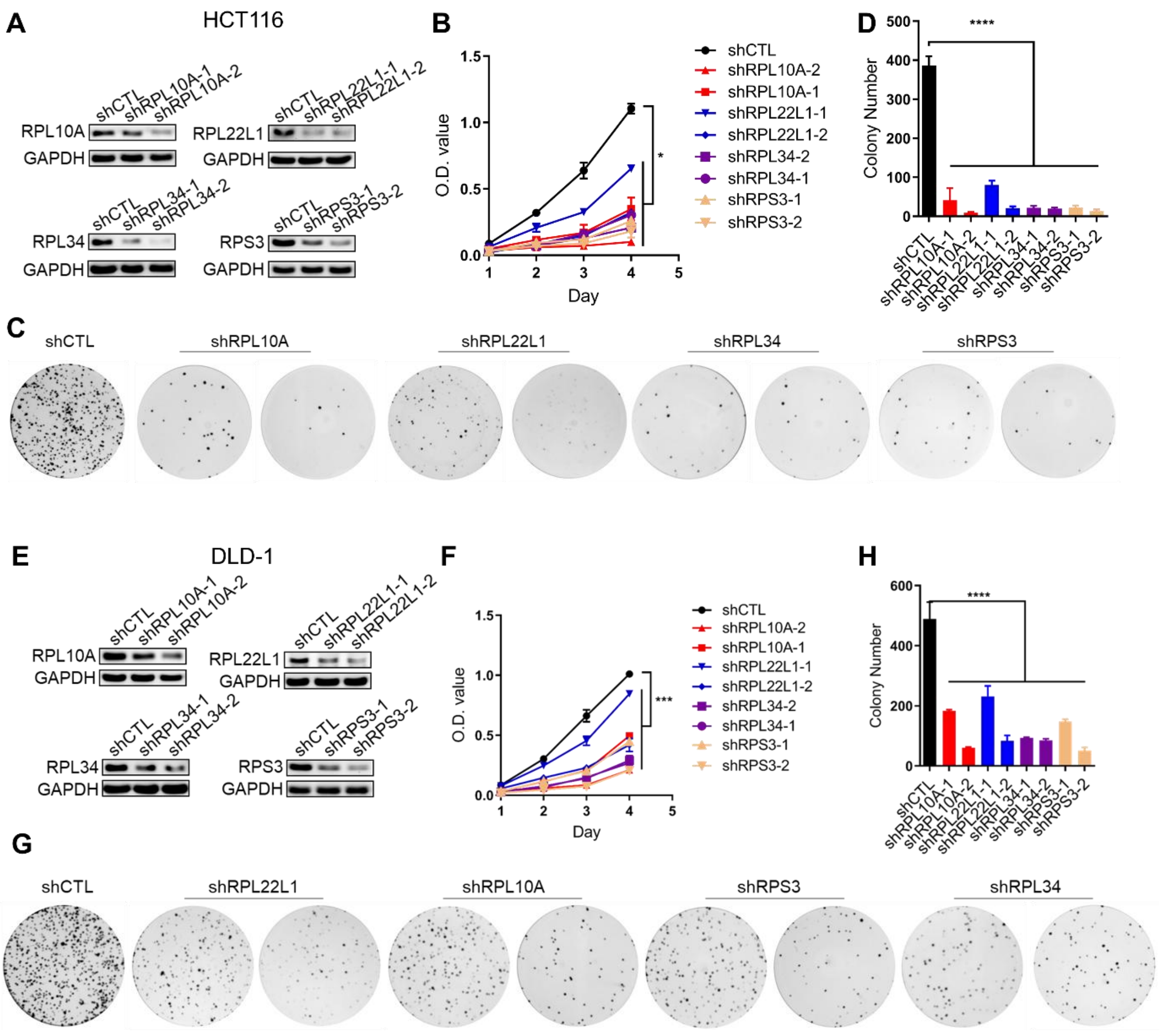

Figure S3. Ribosomal proteins promote colorectal cancer cell proliferation. (**A**) Immunoblot indicating the knockdown efficiency of the four indicated ribosomal proteins in HCT116 cells. (**B-D**) Stable knockdown of each indicated ribosomal protein reduced the growth (**B**) and colony formation (**C-D**) of HCT116 cells. (**E**) Immunoblot indicating the knockdown efficiency of the four indicated ribosomal proteins in DLD-1 cells. (**F-H**) Stable knockdown of each indicated ribosomal protein reduced the growth (**F**) and colony formation (**G-H**) of DLD-1 cells. *P<0.05, ***P<0.001, ****P<0.0001 ((B, F) two-way ANOVA with Bonferroni correction, (D, H) one-way ANOVA with Bonferroni correction).

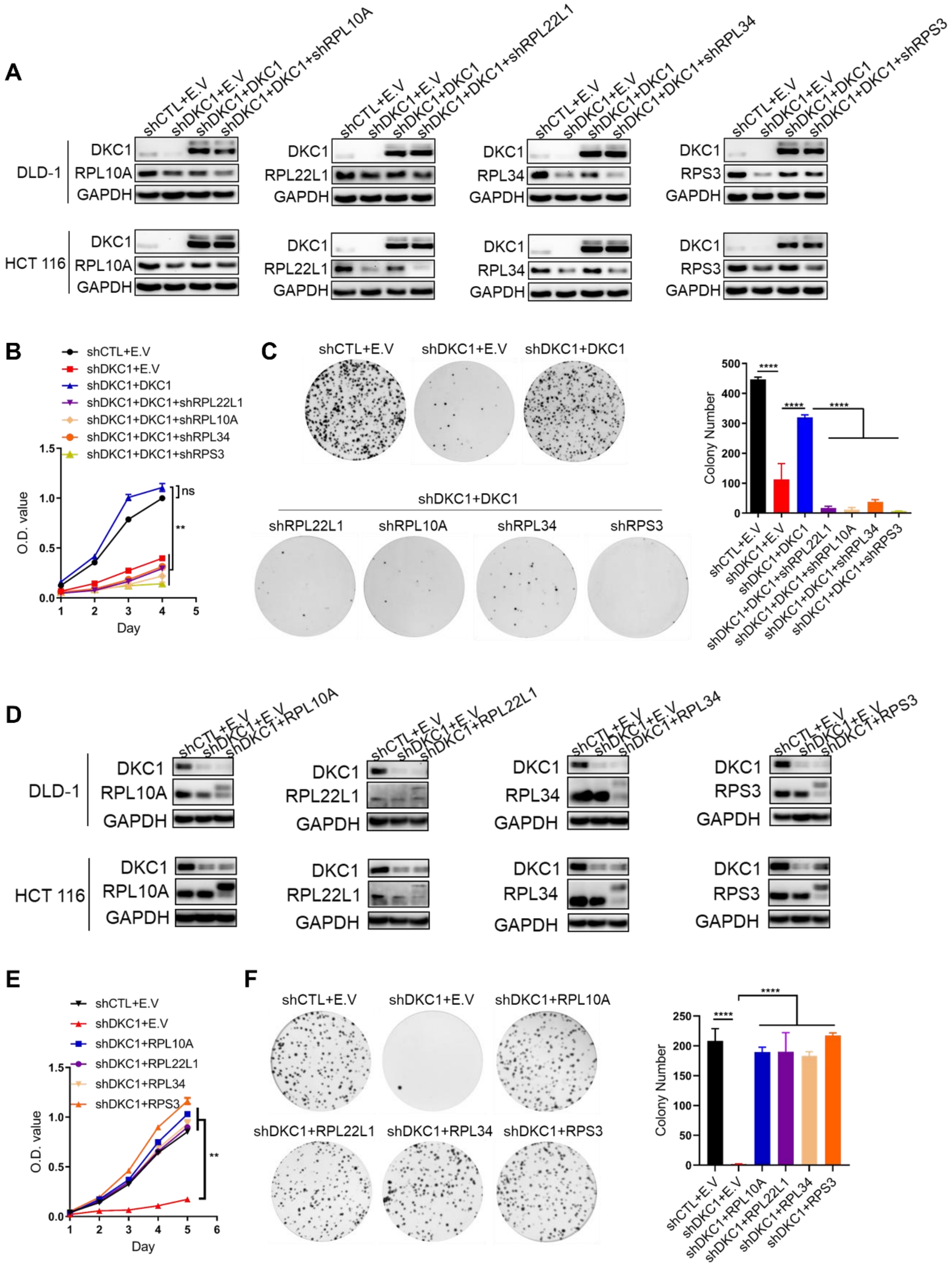

Figure S4. Ribosomal proteins are indispensable for DKC1-mediated acceleration of colorectal cancer progression. (**A**) DLD-1 and HCT116 cells with DKC1 knockdown were rescued with ectopically expressed DKC1 and were further transduced with lentiviral shRNA

individually targeting the four indicated ribosomal proteins. Immunoblotting was performed to detect the expression of the indicated proteins in these cells as well as the corresponding control DLD-1 and HCT116 cells. (**B-C**) HCT116 cells with DKC1 knockdown were rescued with or without ectopically expressed DKC1 and were further transduced with lentiviral vectors carrying shRNA individually targeting the indicated ribosomal proteins. The growth (**B**) and colony formation (**C**) of these cells were assessed. (**D**) The expression of the four ribosomal proteins (RPL10A, RPL22L1, RPL34, RPS3) in DKC1 knockdown DLD-1 and HCT116 cells with overexpression of the indicated genes was assessed by immunoblotting. (**E-F**) HCT116 cells with DKC1 knockdown were transduced with lentiviral vectors individually encoding the indicated ribosomal proteins. The growth (**E**) and colony formation (**F**) of these cells and the control cells were monitored. E.V: empty vector. $^{**}P<0.01$, $^{****}P<0.0001$, ns: no significance ((B, E) two-way ANOVA with Bonferroni correction, (C, F) one-way ANOVA with Bonferroni correction).

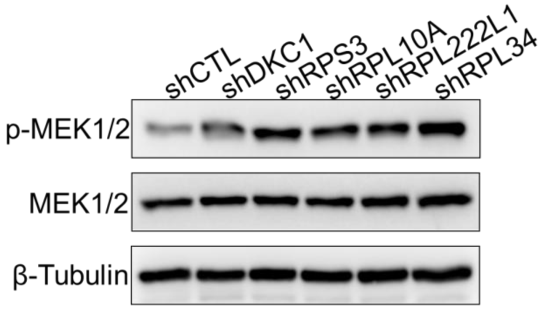

Figure S5. Immunoblot indicating the total expression levels and phosphorylation levels of MEK1/2 in DLD-1 cells with DKC1 or the indicated ribosomal proteins knockdown.

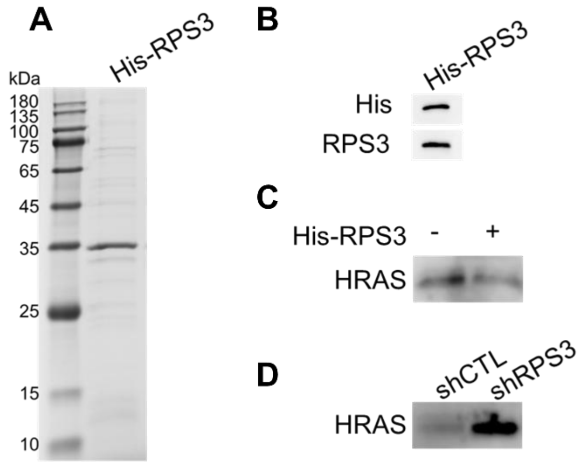

Figure S6. RPS3 inhibits HRAS activation in DLD-1 cells. (**A**) Coomassie blue staining of recombinant human RPS3 fused with His-tag (His-RPS3). (**B**) Immunoblotting was performed to measure purified His-RPS3 expression with anti-His and anti-RPS3 antibodies. (**C**) HRAS activation assay on 3 mg DLD-1 cell lysates with or without His-RPS3 (0.2 μg) (**D**) HRAS activation assay on RPS3 silenced DLD-1 cells or control cells.

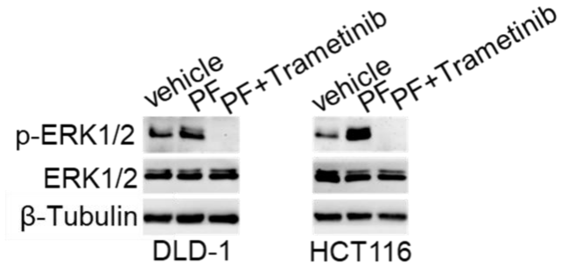

Figure S7. Immunoblot indicating the total expression levels and phosphorylation levels of ERK1/2 in DLD-1 cells and HCT116 cells after PF (0.5 μM) or the combination of PF (0.5 μM) and trametinib (1 μM) treatment for 48 h.

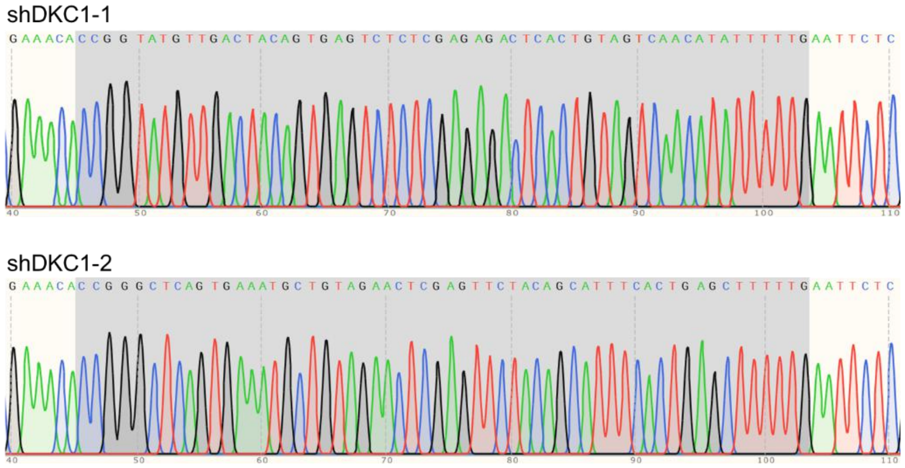

Figure S8. DKC1 shRNAs (shDKC1-1 or shDKC1-2) cloned into pLKO.1 vector were validated by sanger sequencing.

Supplementary Tables

Table S1. 28 differentially expressed ribosomal proteins revealed by proteomics.

**Gene name**	**shDKC1 /shCTL Ratio**	**shDKC1 /shCTL P value**	**shCTL _1**	**shCTL _2**	**shCTL _3**	**shDKC1 _1**	**shDKC1 _2**	**shDKC1 _3**
RPL10A	0.626	0.01498	1.311[§]	1.097	1.295	0.778	0.768	0.772
RPL14	0.513	0.03286	1.185	1.17	1.457	0.745	0.797	0.415
RPL18	0.498	0.00086	1.27	1.282	1.472	0.709	0.706	0.588
RPL22L1	0.24	3.1E-06	1.531	1.585	1.667	0.357	0.408	0.382
RPL24	0.608	0.0014	1.226	1.138	1.365	0.776	0.787	0.706
RPL26L1	0.501	0.00042	1.28	1.356	1.337	0.737	0.66	0.595
RPL34	0.558	0.00052	1.246	1.258	1.351	0.749	0.756	0.645
RPL35	0.606	0.00294	1.19	1.183	1.365	0.789	0.812	0.666
RPL38	0.659	0.00046	1.29	1.204	1.129	0.808	0.793	0.787
RPL7	0.583	3.2E-06	1.261	1.251	1.275	0.728	0.76	0.72
RPL7A	0.59	0.0024	1.239	1.202	1.321	0.789	0.795	0.637
RPS10	0.641	0.00018	1.203	1.253	1.292	0.83	0.81	0.761
RPS11	0.634	0.00022	1.266	1.197	1.22	0.805	0.801	0.73
RPS13	0.652	4.3E-05	1.186	1.194	1.245	0.812	0.787	0.766
RPS16	0.575	0.00108	1.254	1.159	1.396	0.759	0.754	0.677
RPS17	0.636	1.9E-05	1.227	1.189	1.252	0.788	0.76	0.785
RPS18	0.583	1.6E-05	1.219	1.269	1.281	0.746	0.713	0.738
RPS19	0.6	2E-05	1.224	1.251	1.235	0.749	0.765	0.713
RPS2	0.605	2.9E-06	1.221	1.227	1.235	0.746	0.76	0.721
RPS20	0.622	0.00064	1.292	1.21	1.247	0.731	0.848	0.752
RPS26	0.665	0.00176	1.18	1.116	1.257	0.722	0.807	0.832
RPS27A	0.664	0.00112	1.284	1.101	1.142	0.794	0.758	0.79
RPS3	0.66	3.1E-06	1.208	1.204	1.16	0.782	0.789	0.788
RPS4X	0.604	0.0004	1.236	1.186	1.241	0.773	0.766	0.675
RPS5	0.611	2.1E-05	1.262	1.268	1.238	0.782	0.783	0.736
RPS6	0.647	0.00042	1.212	1.246	1.136	0.815	0.776	0.736
RPS8	0.642	6E-07	1.2	1.219	1.238	0.783	0.778	0.785
RPS9	0.573	3.8E-06	1.289	1.255	1.26	0.743	0.734	0.703

[§]Relative expression value of indicated protein.

Table S2. shRNA sequences used in the study.

shRNA	sequence
shDKC1-1 F	5'-CCGGTATGTTGACTACAGTGAGTCTCTCGAGAGACTCACTGTAGTCAACATATTTTTG-3'
shDKC1-1 R	5'-AATTCAAAAATATGTTGACTACAGTGAGTCTCTCGAGAGACTCACTGTAGTCAACATA-3'
shDKC1-2 F	5'-CCGGGCTCAGTGAAATGCTGTAGAACTCGAGTTCTACAGCATTTCACTGAGCTTTTTG-3'
shDKC1-2 R	5'-AATTCAAAAAGCTCAGTGAAATGCTGTAGAACTCGAGTTCTACAGCATTTCACTGAGC-3'
shRPL10A-1 F	5'-CCGGATCAAGCAGATTCCACGAATCCTCGAGGATTCGTGGAATCTGCTTGATTTTTTG-3'
shRPL10A-1 R	5'-AATTCAAAAAATCAAGCAGATTCCACGAATCCTCGAGGATTCGTGGAATCTGCTTGAT-3'
shRPL10A-2 F	5'-CCGGGTCCACAATCAAGTTCCAAATCTCGAGATTTGGAACTTGATTGTGGACTTTTTG-3'
shRPL10A-2 R	5'-AATTCAAAAAGTCCACAATCAAGTTCCAAATCTCGAGATTTGGAACTTGATTGTGGAC-3'
shRPL22L1-1 F	5'-CCGGGAGGTCAACCTGGAGGTTTAACTCGAGTTAAACCTCCAGGTTGACCTCTTTTTG-3'
shRPL22L1-1 R	5'-AATTCAAAAAGAGGTCAACCTGGAGGTTTAACTCGAGTTAAACCTCCAGGTTGACCTC-3'
shRPL22L1-2 F	5'-CCGGCATTGAACGCTTCAAGAATAACTCGAGTTATTCTTGAAGCGTTCAATGTTTTTG-3'
shRPL22L1-2 R	5'-AATTCAAAAACATTGAACGCTTCAAGAATAACTCGAGTTATTCTTGAAGCGTTCAATG-3'
shRPL34-1 F	5'-CCGGGACCTAAAGTTCTTATGAGATCTCGAGATCTCATAAGAACTTTAGGTCTTTTTG-3'
shRPL34-1 R	5'-AATTCAAAAAGACCTAAAGTTCTTATGAGATCTCGAGATCTCATAAGAACTTTAGGTC-3'
shRPL34-2 F	5'-CCGGAGCACAGAGTCAGAAAGCTAACTCGAGTTAGCTTTCTGACTCTGTGCTTTTTTG-3'
shRPL34-2 R	5'-AATTCAAAAAAGCACAGAGTCAGAAAGCTAACTCGAGTTAGCTTTCTGACTCTGTGCT-3'
shRPS3-1 F	5'-CCGGCAGAGTCTCTGCGTTACAAACCTCGAGGTTTGTAACGCAGAGACTCTGTTTTTG-3'
shRPS3-1 R	5'-AATTCAAAAACAGAGTCTCTGCGTTACAAACCTCGAGGTTTGTAACGCAGAGACTCTG-3'
shRPS3-2 F	5'-CCGGTATGGTGTGCTGCGGTTCATCCTCGAGGATGAACCGCAGCACACCATATTTTTG-3'
shRPS3-2 R	5'-AATTCAAAAATATGGTGTGCTGCGGTTCATCCTCGAGGATGAACCGCAGCACACCATA-3'

Table S3. PCR primers used in the study.

Primers	sequence	
DKC1	F: 5'-ATGGCGGATGCGGAAGTAAT-3'	R: 5'-CCACTGAGACGTGTCCAACT-3'
GAPDH	F: 5'-GAAGGTGAAGGTCGGAGTC-3'	R: 5'-GAAGATGGTGATGGGATTTC-3'
RPL10A	F: 5'-TCTCTCGCGACACCCTGTA-3'	R: 5'-GTGGACTTAAGCCTGACGGT-3'
RPL14	F: 5'-TTGGACCTCATGCCGGAAAA-3'	R: 5'-GCACTGTGCGGAAACTTGAG-3'
RPL18	F: 5'-ATGATGTGCGGGTTCAGGAG-3'	R: 5'-AAATGCCGGTACACCTCTCG-3'
RPL22L1	F: 5'-GGCAAAACTGGAAATCTCGGG-3'	R: 5'-GCAACCACTCGAAGCCAATC-3'
RPL24	F: 5'-CGAGCTGTGCAGTTTTAGCG-3'	R: 5'-CTGGAATTTGACTGCTCGGC-3'
RPL26L1	F: 5'-TCACTTCAATGCCCCCTCAC-3'	R: 5'-TCGAACTACCTGGACCTCGT-3'
RPL34	F: 5'-GGGTTCGTGCTGTAAGACCT-3'	R: 5'-AAGCACGCTTGATCCTGTCA-3'
RPL35	F: 5'-CGAGTCGTCCGGAAATCCAT-3'	R: 5'-CAGGTTCTCCTCGTGCTTGT-3'
RPL38	F: 5'-CCGACGAAAGGATGCCAAAT-3'	R: 5'-TTGTCAGTGATGACCAGGGTG-3'
RPL7	F: 5'-GCGAAGGAATTTCGCAGAGC-3'	R: 5'-TTCTTGCCATCCTCGCCATT-3'
RPL7A	F: 5'-CAAAAGAGACCTCACCCGCT-3'	R: 5'-AATCGCAGGAGGCACTTTCA-3'
RPS10	F: 5'-CTGCGAGACTCACAAGAGGG-3'	R: 5'-CACGACCAAATCCGCCTCTA-3'
RPS11	F: 5'-TACCAAAAGCAGCCGACCAT-3'	R: 5'-GCCTCCTTGGGTGTCTTGAA-3'
RPS13	F: 5'-CTGTCCCAGTCGGCTTTACC-3'	R: 5'-GGAGTAAGGCCCTTCTTGGC-3'
RPS16	F: 5'-CAAAGCCCTGGTGGCCTATT-3'	R: 5'-AGGATTTCTGGTAGCGAGCG-3'
RPS17	F: 5'-CAAAACCGTGAAGAAGGCGG-3'	R: 5'-TAATGGCGATCTCCTCGCAC-3'
RPS18	F: 5'-GTGGGCCGAAGATATGCTCA-3'	R: 5'-TGGCTAGGACCTGGCTGTAT-3'
RPS19	F: 5'-GGTTGGCTCCATGACCAAGA-3'	R: 5'-TCCACCATTTTCAGCCCCTC-3'
RPS2	F: 5'-AGAGGCTACTGGGGGAACAA-3'	R: 5'-GCAGTCATCGATACCAGCCA-3'
RPS20	F: 5'-AAGGTTCTAAGACGTGGGATCG-3'	R: 5'-CTTCTCAAAGTGTACTGCTGGC-3'
RPS26	F: 5'-AAGGAACAATGGTCGTGCCA-3'	R: 5'-TGGGAAGCACATAGGCATCG-3'
RPS27A	F: 5'-TGAGACTTCGTGGTGGTGC-3'	R: 5'-ACTCTCGACGAAGGCGACTA-3'
RPS3	F: 5'-CTGGAGTTGAGGTGCGAGTT-3'	R: 5'-CCCTCTGGAAAGCCAAACCT-3'
RPS4X	F: 5'-CAACAGCTTTGCCACTCGAC-3'	R: 5'-TCACCCACTGCTCTGTTTGG-3'
RPS5	F: 5'-ATGCCAAGTACCTGCCTCAC-3'	R: 5'-CGAAGGCATGCTTGACGATG-3'
RPS6	F: 5'-TGTTACTCCACGTGTCCTGC-3'	R: 5'-AAGTCTGCGTCTCTTCGCAA-3'
RPS8	F: 5'-AATACCGTGCCCTGAGGTTG-3'	R: 5'-TACGGTGTGCTGTCGATGAG-3'
RPS9	F: 5'-TGAGGGCAAGATGAAGCTGG-3'	R: 5'-AAGGACGGGATGTTCACCAC-3'
18S rRNA	F: 5'-GTAACCCGTTGAACCCCATT-3'	R: 5'-CCATCCAATCGGTAGTAGCG-3'
RPS3-3′UTR	F: 5'-TGTCATGCTGTGCTCTAAGTGT-3'	R: 5'-AGCCTTATGTGCCTTCAGTGG-3'
